# The AIDS malignancy clinical trials consortium (AMC) patient navigator (PN) initiative

**DOI:** 10.1186/1750-9378-7-S1-P24

**Published:** 2012-04-19

**Authors:** Maria T Botello-Harbaum, Kimberly Mosby, Don Vena, David M Aboulafia

**Affiliations:** 1 AMC Data and Coordinating Center, The EMMES Corporation, Rockville, MD, USA; 2 Division of Hematology-Oncology, Virginia Mason Medical Center, Seattle, WA, USA; 3 The Division of Hematology, University of Washington, Seattle, WA, USA

## Background

Cancer remains a major health concern in the management of HIV infection in areas of the world with and without access to highly active antiretroviral therapy. In the USA alone, approximately 56,000 people were newly diagnosed with HIV infection in 2006; 53% of these new diagnosis occurred in gay and bisexual men but Black/African American men (45%) ,women (27%) , and Hispanics (17%) were also strongly affected [1]. Recruitment of members of these groups into cancer clinical trials has traditionally been challenging [2]. Among domestic AMC studies, a relatively small percentage of all participants have included women (8%), African-Americans (29%), and Hispanics (21%). Patient Navigation has been identified as an effective strategy to reduce barriers to care as well as to increase access to cancer clinical trials [3]. In an effort to bolster opportunities for HIV-infected minorities, women, and medically underserved populations to become involved in AMC clinical trials, a PN initiative was implemented in seven AMC sites located in Boston, Los Angeles, San Diego, Houston, Columbus, and Honolulu. The main objectives of the PN initiative were to provide greater opportunity for minority groups and women to participate in AMC-sponsored cancer trials and to increase awareness of HIV/AIDS malignancies in the local communities where the PNs worked. From January 2010 to April 2011, PNs implemented multi-strategy activities to increase the enrollment of women and minorities in AMC trials. PNs reported 466 activities in the programmatic areas of recruitment and retention, community outreach and education and awareness. Recruitment and retention refers activities to recruit new participants and increase retention in AMC trials. Community outreach was targeted to the medical community or the general population to increase their awareness of AIDS-related malignancies. Education and awareness were activities to educate the community on HIV-related malignancies in general and AMC-sponsored clinical trials in specific. PNs efforts were concentrated on community outreach (54%, n=251), followed by recruitment and retention (28%, n=129) and education and awareness 18% (n=86).

## Conclusion

AMC-PNs conducted activities that raised awareness in their local communities of AIDS-related malignancies, developed partnerships with local health community organizations and identified areas where further communication was needed. PNs took the lead in developing a PN brochure and in the design of several tailored recruitment strategies. The PN program is making important inroads into behavioral interventions to increase participation of minorities and underserved populations in AMC trials.
